# Advances in self-healing supramolecular soft materials and nanocomposites

**DOI:** 10.1186/s40580-019-0199-9

**Published:** 2019-08-15

**Authors:** Gurunathan Thangavel, Matthew Wei Ming Tan, Pooi See Lee

**Affiliations:** School of Materials Science and Engineering, Nanyang Technological University, Singapore, 639798 Singapore

**Keywords:** Self-healing, Supramolecular polymer nanocomposites, Hydrogen bonding, Metal-complexes

## Abstract

The ability to rationally tune and add new end-groups in polymers can lead to transformative advances in emerging self-healing materials. Self-healing networks manipulated by supramolecular strategies such as hydrogen bonding and metal coordination have received significant attention in recent years because of their ability to extend materials lifetime, improve safety and ensure sustainability. This review describes the recent advancements in supramolecular polymers self-healing networks based on hydrogen bonding, metal-containing polymers and their nanocomposites. Collectively, the aim of this review is to provide a panoramic overview of the conceptual framework for the interesting nexus between hydrogen bonding and metal–ligand interactions for enabling supramolecular self-healing soft materials networks and nanocomposites. In addition, insights on the current challenges and future perspectives of this field to propel the development of self-healing materials will be provided.

## Introduction

Self-healing in living organisms has played an essential role in sustaining and prolonging life. In contrast, synthetic materials are often susceptible to degradation, fracture or damage by external factors, leading to a limit to its lifetime [1–5]. Inspired by nature, a significant amount of research has been dedicated towards self-healing materials that are able to restore its fundamental properties including mechanical and electrical functions after damage [6]. In particular, self-healing polymers have garnered much interests due to its role in a broad range of applications, such as electronics, transportation, and medicine. With these self-healing abilities, significant improvements can be brought towards extending the functional lifetime, reliability, energy efficiency and product safety of polymer materials [7–10]. Furthermore, with growing concerns over pollution from environmental waste, self-healing polymers play a critical role in addressing these issue.

Self-healing polymers can be classified into two categories—intrinsic and extrinsic. Extrinsic self-healing relies on two components. First, micro-containers of healing agents that may be in the form of microcapsules [4], or hollow fibers [11] and second, a catalyst dispersed within the polymer matrix. Upon mechanical damage, self-healing can be initiated as micro-containers upon ruptured, causing healing agents to be released into the cracks via capillary motion. When the released healing agents come into contact with the dispersed catalyst, polymerization takes place via catalytic crosslinking, re-bonding happens on the crack sites [12]. While extrinsic self-healing methods allow instantaneously healing and are closer to commercialization as no changes to the molecular structure are required; they can only be utilized once or limited amount at each location because of the exhaustion of the micro-containers [13]. Therefore, to overcome this limitation, intrinsic self-healing has often been sought after, as they have been designed with the ability to sustain multiple healing cycles.

**Fig. 1:**
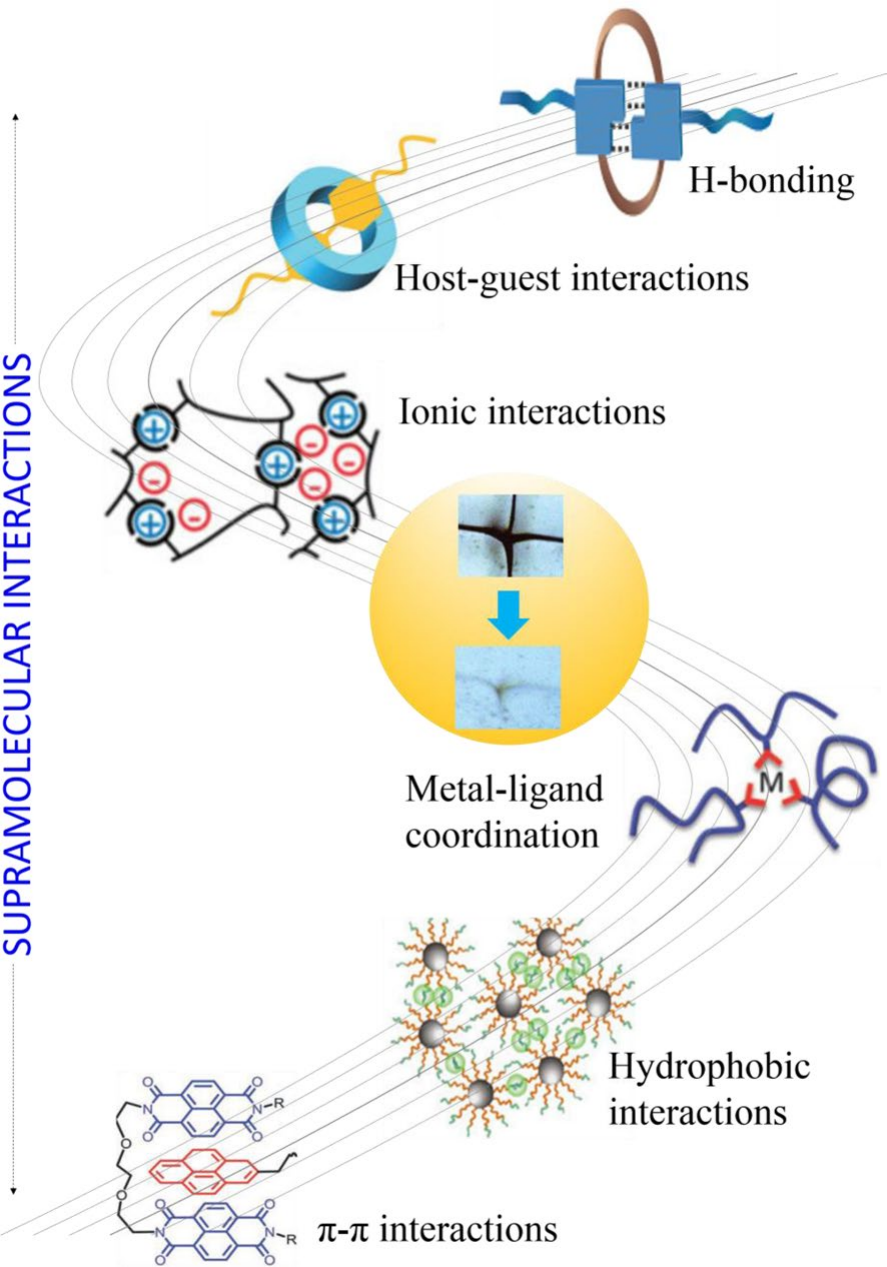
Chemical strategies utilizing supramolecular chemistries for the design of self-healing functional materials, including dynamic covalent bonds and non-covalent interactions (Self-healing optical images: reproduced with permission from: Ref. [90], ©2018 AAAS. Host–guest, metal-coordination and π–π interactions images: reproduced with permission from: Ref. [59], ©2018 WILEY)

Intrinsic self-healing relies on the presence of inherent dynamic bonds incorporated into the polymer, allowing it to sustain several healing cycles (break/reform) [14]. The term dynamic bonds can be further classified into two groups—dynamic covalent bonds and supramolecular interactions. Dynamic covalent bonds encompass a wide range of re-bonding mechanisms such as Diels–Alder and retro-Diels–Alder reactions [15], trithiocarbonate reshuffling reactions [16], triazolinedione-based click [17], and dynamic urea bonds [18]. However, the dissociation and re-association of these self-healing processes generally require external stimulus interventions, such as heat [19], redox [20, 21], light [22], electricity [23], and ultrasound to trigger these reversible reactions [24, 25]. In contrast, intrinsic self-healing based on supramolecular interaction is able to achieve autonomic self-healing. In general, these supramolecular interactions refer to reversible noncovalent bonds such as H-bonding [26], ionic interactions [27], metal–ligand coordination [28], host–guest interaction [28], and π–π stacking or hydrophobic interactions [29]. The basic self-healing chemistries are depicted in Fig. 1, demonstrating major strategies for designing self-healing supramolecular polymers. Owing to the weak nature of these supramolecular interactions, the non-covalent bonds between polymer chains are primarily broken upon mechanical damage. However, these bonds easily reform with each other after being broken, enabling autonomous self-healing to occur [1].

Over the years, several notable works on autonomous supramolecular self-healing polymeric materials have been reported [30]. The importance of chain mobility and dynamics for self-healing has been well illustrated by Sun et al. [31]. Meijer and coworkers were the first to utilize 2-ureido-4[1*H*]-pyrimidinone (UPy) monomers for quadruple hydrogen bonding (H-bonding) noncovalent systems with a high degree of polymerization [32]. Guan et al. [33] have reported supramolecular polymer based on UPy repeating units, and showcased a rare combination of high-level toughness, high strength, shape memory, and self-healing.

With the potential of supramolecular interactions to provide self-healing that is both autonomous and able to undergo several healing cycles, the advancement of supramolecular chemistries, described by Lehn as the “chemistry beyond the covalent bond” has induced a convergence between materials science and supramolecular chemistry [34]. This can be clearly represented from the application of supramolecular interactions to nanocomposite materials [35]. Through the exploitation of these chemistries, reversible non-covalent interactions and polymeric structures of supramolecular polymer systems endow traditional covalent polymers with dynamic unique properties to achieve self-repairing capacities. This review delineates the recent developments of supramolecular self-healing functional polymeric materials; in which we will focus on two critical supramolecular chemistries relevant to self-healing interactions: hydrogen bonds and metal–ligand interactions. These strategies to manipulate the macromolecular structure and design of supramolecular polymers will be discussed in detail, followed by the implementation of these strategies on polymer nanocomposites. Finally, a perspective on the challenges for these self-healing materials systems will be examined.

## Hydrogen-bonded supramolecular self-healing

Hydrogen bonding is a directional dipole–dipole attraction between an electronegative atom and a hydrogen atom bonded to another electronegative atom with a lone pair of electrons, such as, oxygen, nitrogen or fluorine. The bond energy of hydrogen bonds falls within the range of 5 to 30 kJ mol^−1^, undoubtedly lower compared to carbon–carbon (C–C) bonds (~ 345 kJ mol^−1^ for covalent bonds) [34]. Nonetheless, despite being approximately 10 times weaker, the presence of hydrogen bonding greatly influences the bulk viscoelasticity, the degree of phase separation and the degree of crystallinity of materials [36]; arising from its directionality and affinity. In general, polymer network rearrangement and mechanical properties can be tuned by controlling the concentration and strength of hydrogen bonds as well as the polymer’s backbone rigidity [37]. Taking these factors into consideration, various strategies utilizing hydrogen bonding for supramolecular self-healing will be discussed at greater detail in this section, including the usage of carboxyl and UPy moieties. Table 1 summarizes recent self-healing hydrogen-bonded supramolecular polymers, specifying their mechanical characteristics, healing motifs, and healing conditions.

**Table 1:** Summary of recent self-healing systems based on supramolecular hydrogen bonds and metal-coordination crosslinking: mechanical characteristics, self-healing-conditions, and efficiencies

Polymer matrix	Healing motif	*T*_g_ (°C)	Mechanical characteristics		Healing conditions	Healing efficiency	Appearance	Ref.
			UTS (MPa)	ES (%)				
HBP (PS/PA-amide)	H-bonding	2–5	1.9	780	RT, 24 h	90%	Non-transparent	[26]
FA and urea	H-bonding	28	3.4	600	RT, 18 h	80%	Non-transparent	[92]
BCP	H-bonding	~ 2–4	~ 4.38	~ 750	60 °C, 24 h	~ 90%	Non-transparent	[94]
SPB-2%	H-bonding	1	1.02	410	RT, 1 h	95%	Transparent	[95]
HN-DGEBA-TGMDA	H-bonding	23	1.5	325	RT, 24 h	~ 100%	Non-transparent	[96]
PMMA, PA-amide	H-bonding	1–5	3	500	RT, 24 h	80%	Non-transparent	[97]
Crosslinked PU	H-bonding	− 7	~ 26.5	~ 870	100 °C, 24 h	92.3%	Non-transparent	[98]
TUEG_3_	H-bonding	27	45	393	140 °C, 30 s	~ 85%	Non-transparent	[58]
LPU	H-bonding	16–40	1.57	104.87	50 °C, 60 min	96%	Transparent	[99]
Fe-Hpdca-PDMS	Metal–ligand	90	0.03	1850	RT, 48 h	90%	Non-transparent	[28]
Fe-triazole-PDMS	Metal–ligand	− 90	0.22	3400	60 °C, 20 h	94.3%	Non-transparent	[100]
Zn(OTf)_2_-PDMS	Metal–ligand	− 50	0.63	330	RT, 48 h	76%	Non-transparent	[101]
Co-triazole-PDMS	Metal–ligand	− 100	1.12	560	140 °C, 24 h	52.2%	Non-transparent	[102]
HBN-1% GO	Metal–ligand	− 5 to 9	0.5	550	RT, 1 h	~ 100%	Non-transparent	[103]
ACON	Metal–ligand	− 5 to 40	2	920	50 °C, 3 h	83%	Non-transparent	[104]
MD_50_ -F_5_	Metal–ligand	18 to 64	12	450	90 °C,12 h	98%	Non-transparent	[105]
ICPs-Zn (NTf_2_)_2_ (ICP-2)	Metal–ligand	− 37.8	1.7	593	RT, 3 h	~ 100%	Non-transparent	[63]

UTS: ultimate tensile strength; EB: elongation at break; *T*_g_: glass transition temperature obtained from DSC/DMA; Fe-Hpdca-PDMS-Fe-2,6-pyridinedicarboxamide (pdca) coordination complex with PDMS; Zn(OTf)_2_-PDMS-Zinc trifluoromethanesulfonate-PDMS based metal–ligand coordination; HBN: amine-terminated randomly branched oligomer; GO: graphene oxide; SPB-2%: the functionalized polybutadiene-COOH and polybutadiene-NH_2_ based on ionic hydrogen bonding with 2 wt% of tri-functional thiol as a covalent cross-linker; DGEBA: a bifunctional diglycidyl ether of bisphenol A; TGMDA: a tetrafunctional 4,4′-methylenebis (*N*,*N*-diglycidylaniline); HN-50_75%DGEBA_25%TGMDA: hybrid networks containing 75% DGEBA and 25% TGMDA; ACON: secondary amide-containing cyclooctene (CO) network via carbodiimide coupling with *N*-acetylglycine; PMMA-PA amide: a hard polymethylmethacrylate (PMMA) and soft polyacrylate-amide (PA-amide) brushes that exhibit thermoplastic elastomer properties; MD_50_-F_5_: poly-*N*,*N*-dimethylacrylamide-co-2-methoxyethyl acrylate + 5% Fe_2_O_3_; crosslinked PU: the amount of the synchronous (C-ON) bond involved in fission/radical recombination that enables interrelated reprogramming, intrinsic self-healing of wider crack and recycling of the crosslinked PU; TUEG_3_: poly(ether-thioureas) with triethylene glycol; LPU: a linear polyurethane with high contents of urea and urethane group for h-bonding formations to facilitate self-healing; ICPs-Zn(NTf_2_)_2_: imidazole-containing brush polymers (ICPs)-zinc di[bis(trifluoromethylsulfonyl)-imide] (Zn(NTf_2_)_2_) based metal–ligand (zinc-imidazole) interactions in the soft matrix of a hard/soft two-phase brush copolymer system and BCP: block copolymers (PA-amide)-b-PMMA-b-(PA-amide)

### Carboxylated-bonded supramolecular self-healing

**Fig. 2:**
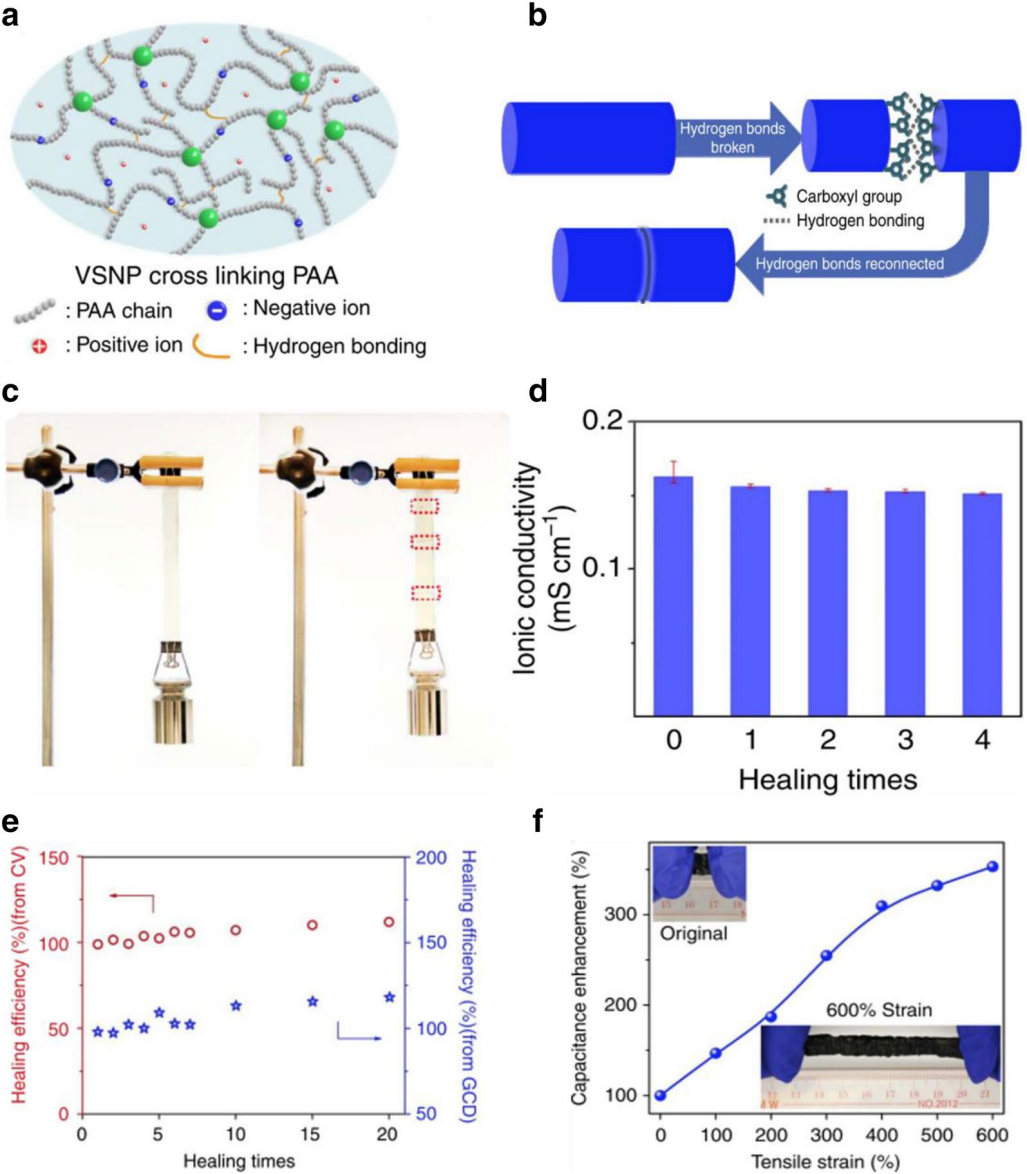
**a** Schematic of the H-bonding and vinyl hybrid silica nanoparticles (VSNPs) dual cross-linked VSNPs-PAA electrolyte, which are intrinsically neither self-healable nor highly stretchable; **b** schematic of self-healing arising from interfacial coordination of carboxyl groups on the PAA. **c** Demonstration of the self-healed VSNPs-PAA polyelectrolyte film (3.4 mm thick × 1.8 cm wide) to support ca. 500 g mass: pristine sample (left of panel) and healed sample after the third breaking/healing cycle (right of panel); **d** ionic conductivity of the VSNPs-PAA polyelectrolyte after multiple healing cycles; **e** after healing from different cycles, the healing efficiency calculated from CV (circle, red) and GCD (star, blue) curves; **f** capacitance calculated from the GCD as a function of the tensile strain achieves an increase of 3.5-and 2.1-fold at 600% strain (reproduced with permission from: Ref. [38] ©2015, NPG)

**Fig. 3:**
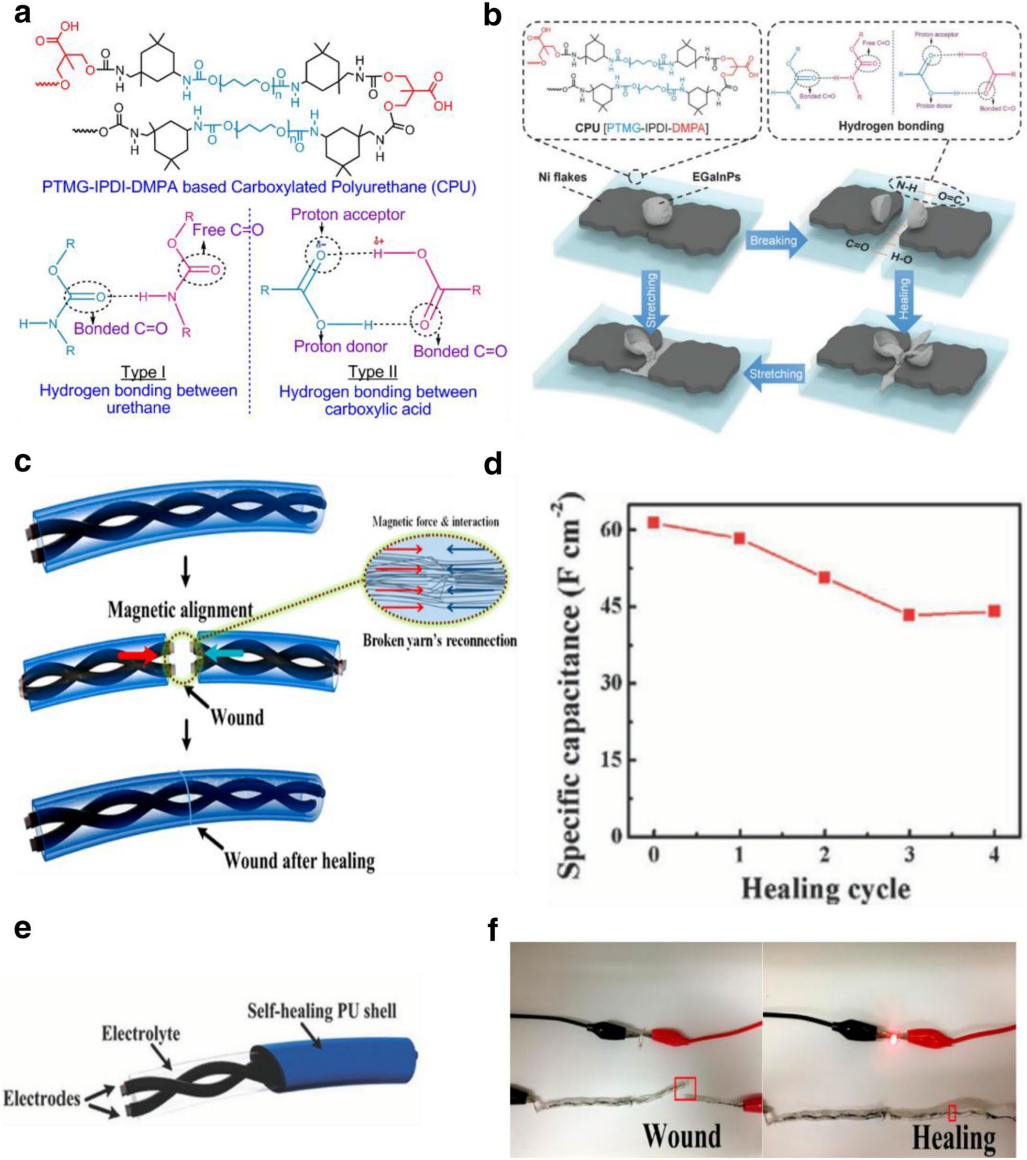
**a** Schematics of the synthesis and hydrogen bonding mechanism of the CPU, wherein various stronger –COOH and weaker –NH–CO–O crosslinks are introduced to tune the mechanical properties of CPU; **b** schematics representation of mechanically and electrically self-healable and stretchable Ni flakes–EGaInPs–CPU conductor. The conductor electrically restored and mechanically healing activated due to the interfacial H-bonding of CPU with an efficiency of 97.5%; **c** schematic illustration of the self-healing process of supercapacitors. The demonstrated image shows the performance after self-healing on the fractured surfaces is fully based on the synergistic effect of magnetic electrodes and the CPU shell; **d** specific capacitance of the original device and after four breaking/healing cycles, which is restored to 71.8% due to the interfacial CPU shell. **e** Schematic structure of the magnetic-assisted self-healable yarn-based supercapacitor; **f** photographs of a self-healing supercapacitors connected in series to power an LED bulb (figures reproduced with permission from: **a**, **b** Ref. [59], ©2019 WILEY; **c**–**f** Ref. [61], ©2015 ACS)

Carboxyl groups have often been utilized to form supramolecular motifs to induce self-healing properties. Apart from forming a single hydrogen bond, the presence of hydroxyl and carbonyl groups enables the formation of carboxyl cyclic dimers as these groups can bind cooperatively as they can act as both proton donors and proton acceptors at the same time (Fig. 3a). The role of carboxyl groups in self-healing can be clearly demonstrated from Zhi’s group who fabricated a polyelectrolyte comprising of polyacrylic acid (PAA) dual crosslinked by H-bonding and vinyl hybrid silica nanoparticles (VSNPs-PAA) (Fig. 2) [38]. This polyelectrolyte showed high stretchability, self-healability and ionic conductivity (Fig. 2f). Superior self-healing abilities of the electrolyte arises from the assembly of carboxylated groups present in VSNPs-PAA (Fig. 2b), allowing hydrogen bonds to reform after being bisected. This was clearly demonstrated with supercapacitors using VSNPs-PAA polyelectrolyte films that showed nearly ~ 100% efficiency in retention of its capacitance during all 20 breaking/healing cycles (Fig. 2e), significantly out-performing other self-healing supercapacitors. Furthermore, the incorporation of wavy electrodes allowed the fabrication of a highly stretchable supercapacitor that achieved up to 600% strain.

Carboxyl groups have often been used in conjunction with polyurethanes (PU). With the addition of these motifs, polyurethanes can be specially designed to exhibit self-healing abilities in addition to its versatile structure and performance. This unique property is attributed to the increase in hydrogen bonds, at which supramolecular arrays of molecules are held together by dimerization of carboxylic acid group to have a high degree of polymerization [39]. This effect is well illustrated by Hu’s group at which polyurethane without carboxyl groups cannot be healed. On the other hand, with the addition of carboxyl groups, self-healing polyurethane was achieved with the increased number of hydrogen bonding [40]. In some cases, the hydrogen bonds between carboxylic acids can also result in a stabilized shape memory complex [41].

Earlier, our group described a highly stretchable (1000%) and printable conductors of silver flakes, EGaInPs, and ethylene–vinyl-acetate (EVA) polymer matrix for soft touch sensor arrays [42]. This work was further extended by introducing carboxylated polyurethanes (CPUs) as the polymeric matrix to achieve self-healing stretchable conductors (Fig. 3) [43]. The versatility of these self-healing conductors was demonstrated through its application as an energy storage device [43]. For superior stretchability with room temperature self-healable energy storage purpose, various amounts of strong carboxylate H-bonding and weak (–NH–CO–O–) bonds were introduced to the backbones of polymers to tune the tensile properties. Here, CPU is composed of a hard domain (high *T*_g_) and a soft brush (low *T*_g_) that impart more H-bonding sites within the supramolecular linkages. Such supramolecular self-assembly (large eight-membered ring containing two H-bonds) and low *T*_g_ of the CPU allow polymer chains to reversibly break and reform spontaneously (Fig. 3). To provide mechanical–electrical restorations simultaneously, conductive liquid–metal (LM), Ni flakes with eutectic-gallium-indium particles (EGaIn) were embedded within the CPU matrix [43]. Upon extreme stretching, fresh EGaIn was released from the gallium oxide shell and filled the cracks to instantly restore the conductivity of the cracked pathway as shown in Fig. 3b. The self-healed stretchable conductor showed an initial conductance of 2479 Scm^−1^ at 700% strain with a 100% recovery of its stretchability and 75% recovery of its electrical properties.

Other applications of carboxylated polyurethane include its use as a self-healing outer shell for various supercapacitors ensuring mechanical self-healing to recover the configuration integrity and mechanical strength [44, 45]. For example, a magnetic-assisted self-healing fiber/yarn-shaped supercapacitor was designed and fabricated by wrapping magnetic electrodes in a self-healing CPU shell (Fig. 3c) [45]. The magnetic electrode was fabricated by depositing Fe_3_O_4_ nanoparticles and protecting materials polypyrrole (PPy) on the stainless steel yarns (Fig. 3e). Once the supercapacitor electrodes are subjected to mechanical damage, strong magnetic force build by Fe_3_O_4_ particles could guide the reconnection of the broken yarn electrodes, leading to the self-healing of electrical conductivity (Fig. 3f). The external movement of the self-healing CPU shell could realize. Due to the synergistic effects of magnetic electrodes and healable PU shell, the specific capacitance is restored to 71.8% even after four breaking/healing cycles (Fig. 3d).

While the carboxyl group offers a wide range of possibilities, the endowed self-healing capability becomes obsolete at high pH. When these groups are exposed to these environments, deprotonation occurs, causing the hydrogen bonds to be hindered due to electrostatic repulsion [46]. However, this issue could be addressed with the inclusion of zwitterionic groups to achieve self-healing in these conditions.

### UPy-bonded supramolecular self-healing

Single hydrogen bonds are insufficient to induce supramolecular assemblies. However, this can be overcome with the inclusion of multiple hydrogen bonds that expand both directionality and versatility, building stronger binding affinities. These multiple hydrogen bonds are arrayed to create hydrogen bonding arrays in which the bonding strength is dependent on the position of the hydrogen donor and acceptor [37]. Furthermore, to achieve supramolecular assemblies with a high degree of polymerization, multiple hydrogen bond arrays with high binding constants are being selected. This has led to several studies that highlighted supramolecular self-healing polymer based on UPy motifs in recent years. Extensive work by Meijer, Zimmerman, and Sijbesma demonstrates the self-healing capabilities of supramolecular polymers upon the introduction of UPy motifs [47–49].

**Fig. 4:**
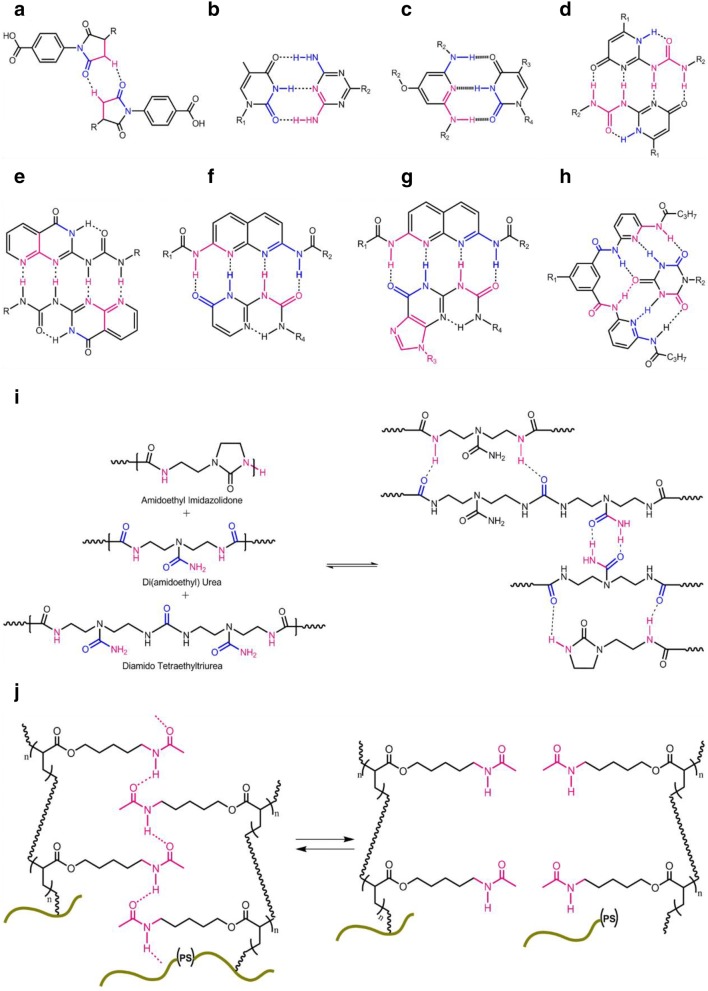
Hydrogen bonding responsible for self-healing properties into supramolecular polymers: **a** two phenyl urazole acids; **b** thymine/2,6-diamino-pyridine (THY/DAP); **c** thymine/diamino triazine (THY/DAT); **d** self-complementary UPy quadruple hydrogen bonding unit (dimerization constant of *K*_dim_ = 10^−7^–10^−8^ M^−1^ and a lifetime of 0.1–1 s); **e** 2-amino-3*H*-pyrido-(2,3-d)pyrimidin-4-one; **f** UPy and 2,7-diamido-1,8-naphthyridine (DAN) (UPy-DAN); **g** 2,7-diamido-1,8-naphthyridine (DAN) and ureido-7-deazaguanine (DeUG) (DAN-DeUG); **h** Hamilton wedge and cyanuric/barbituric acid wedge (HW/Ba); **i** low molecular weight building blocks based on a mixture of fatty diacid and triacid, form a complementary H-bonding; **j** brush copolymer consisting of a hard polystyrene (PS) backbones with soft polyacrylate amide (PAA) pendant groups shows dynamic microphase separation character due to the amide (PAA) hydrogen bonds and sufficient chain mobility (reproduced with permission from: **a**, **d**–**h** Ref. [91], ©2015 Springer; **b** Ref. [41], ©2014, ACS; **c** Ref. [50], ©2010, ACS; **i** Ref. [92], ©2008 NPG; **j** Ref. [26], ©2012 NPG)

**Fig. 5:**
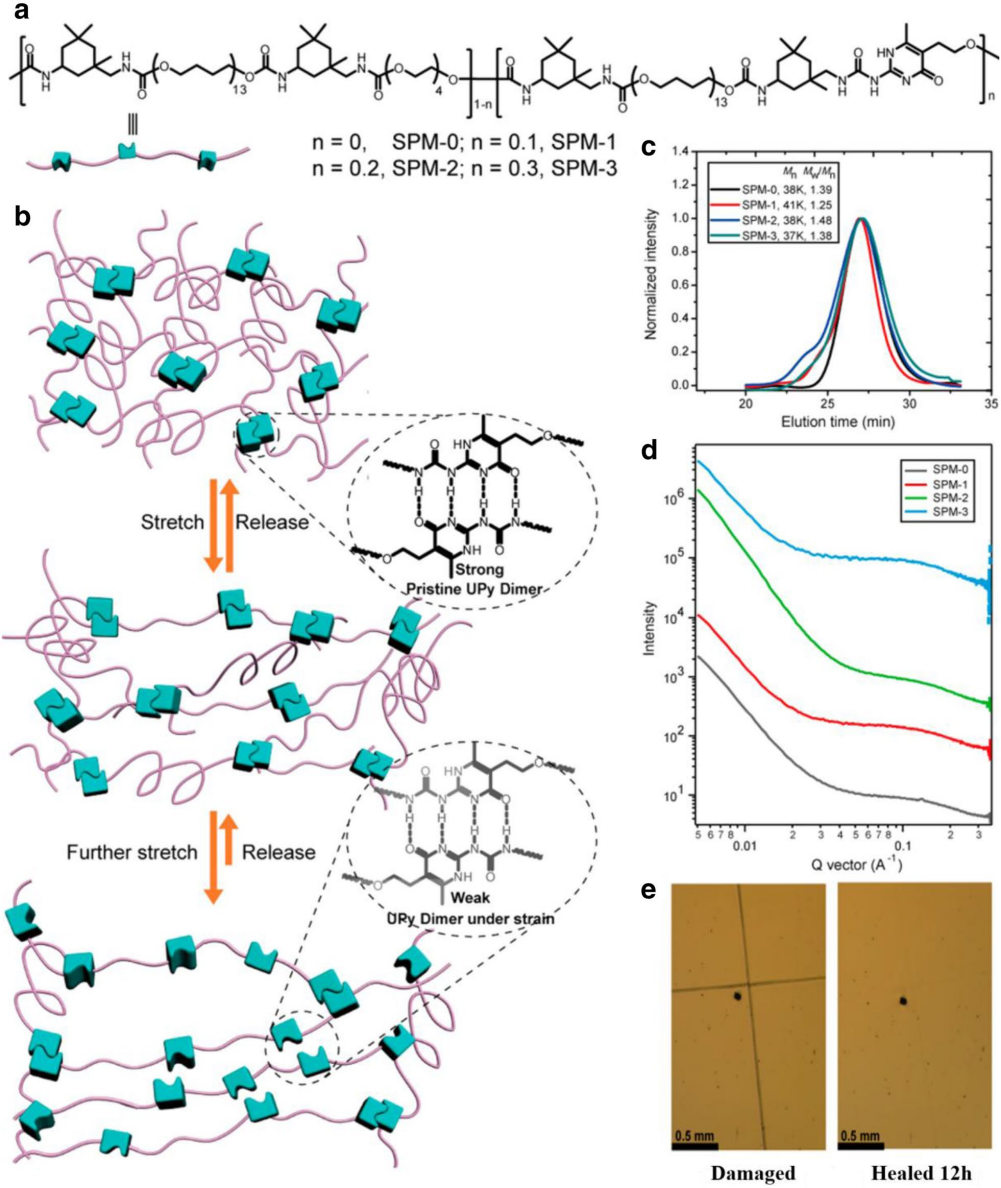
**a** Chemical structures of supramolecular polymeric materials (SPMs) with different amounts of stronger UPy quadruple H-bonding and weaker urethane cross-links to tune the properties of SPMs; **b** schematic illustration of the construction of highly stretchable SPMs; **c** GPC chromatograms utilizing DMF as the eluent and PMMA as the standard, showing that all polymer are comparable *M*_n_ and relatively narrow PDI, respectively; **d** SAXS profile of bulk SPMs that displays broad scattering peaks at 3.7 to 5.2 nm, indicative the presence of microphase separation; **e** optical images of the damaged and healed SPM film (reproduced with permission from: Ref. [52], ©2018 ACS)

UPy is an attractive quadruple-hydrogen-bonding array with a high degree of polymerization (DP), binding strength and synthetic accessibility [50]. When compared with associative groups with trivalent hydrogen bonding systems, such as thymine (THY)/2,6-diamino-pyridine (DAP) interactions (with binding constant *K*_a_ ranges from 10^2^ to 10^5^ m^−1^) (Fig. 4b), quadruple self-complimentary hydrogen bonds such as in UPy showed superior *K*_a_ of 10^7^ m^−1^ (Fig. 4d) [37, 51]. Yan et al. [52] reported a supramolecular polymer composed of isophorone diisocyanate (IPDI), poly(tetramethylene ether) glycol (PTMG) and tetraethylene glycol (TEG) segments that displayed high stretchability (~ 17,000%), high toughness (fracture energy ~ 30,000 J/m^2^), and enhanced autonomous self-healing through the inclusion of self-complementary UPy quadruple H-bonding cross-linked networks (Fig. 5a, b). It was hypothesized that the high stretchability originated from the high binding energy of UPy motif that led to partial breakage or allowed the broken UPy units to be closer to its adjacent chains; leading to dimerization and the formation of weak temporary crosslinks when strained. Furthermore, self-complementary hard UPy crosslinks in the supramolecular polymer had negligible influence on the *T*_g_ of the soft PTMG segments allowing the polymer to be soft, stretchable yet tough. Interestingly, in this work, the unique self-healing property offered by UPy motifs allowed both electrical and mechanical restoration where the self-healable stretchable thin film electrode remained conductive under 90% strain after being healed. The thin metal film covering a cut and bent surface was able to reconnect as the substrates upon healing (Fig. 5e).

It has been well established that water acts as a strong competitor for hydrogen bonding and cause dissociation to the hydrogen bond interactions. To address this, hydrophobic functional groups on siloxane have often been utilized to shield these hydrogen bonds from water [53, 54]. Interestingly, Liu et al. [54] utilized water for enhanced self-healing through multiphase-assembled siloxane oligomeric network UPy terminated tri-functional oligomers ((UP)_3_T) with UPy motifs, amino-terminated short-chain poly(dimethylsiloxane) (PDMS) and tri-functional homo-polymer of hexamethylene diisocyanate. (UP)_3_T was found to be strong and stiff as a result of the dimerization of UPy motifs while the low permeability of water molecules in the hydrophobic PDMS phases ensured that water molecules could pass over the (UP)_3_T network to exchange with UPy motifs but shorten the chain relaxation process. Here, water played a key role as a plasticizer through binding with UPy motifs but was complemented with PDMS phases that prevented uncontrollable dissociation and collapse of the oligomer assembly. The resulting polymer showed unique water-enhanced healing and high toughness due to the enhanced dimerization of UPy motifs and the clustering of the UPy dimers. This water enhanced self-healing ability, can be seen by comparing the recovery of mechanical strength in air (RH ~ 35%), reaching 5.46 MPa for 70 °C at 12 h. The (UP)_3_T polymer film is also self-healing underwater, where the self-healing process is accelerated naturally: tensile properties of healed films are recovered by 86% at 20 °C underwater for 4 days.

Folmer et al. discovered a new synthetic strategy for coupling reactions of UPy moieties with various hydroxytelechelic polymer chains, such as polycarbonates, polyethers, and polyesters, which can be further connected with other polymer chains to form supramolecular block copolymers [47, 55]. The addition of UPy monomer results in thermoplastic elastomeric behavior of copolymers and the resultant materials associate the conventional polymers with the low melt viscosity. OH-telechelic based poly(ethylene-co-butylene) (PEB), a completely amorphous and viscous liquid, when functionalized with UPy units becomes an elastic solid. The functionalized polymer of polycarbonate and polyester are semi-crystalline, whereas the hydroxy-telechelic or UPy-telechelic polycaprolactone (PCL) are brittle solids.

**Fig. 6:**
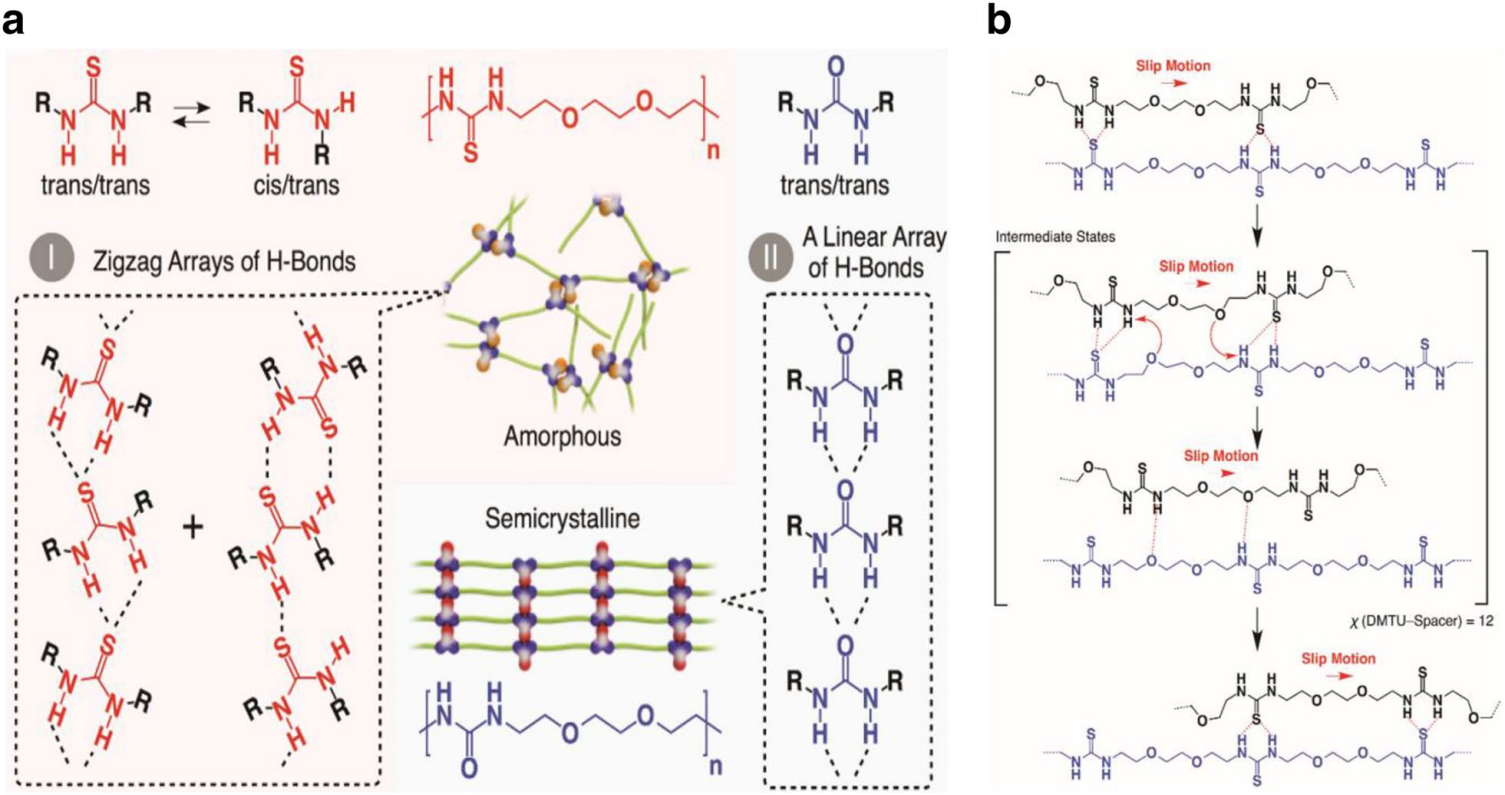
H-bonding approach of thiourea and urea moieties: **a** thiourea units which form nonlinear zigzag arrays adopting cis/trans and strained trans/trans conformations (I), whereas urea groups form linear arrays of H-bonding adopting only a trans/trans conformation (II); **b** proposed mechanism for transformations of H-bonded thiourea pairs. The triethylene glycol spacer that connects the thiourea units optimally modulates the exchange of H-bonded thiourea pairs; consequently, the highly crosslinked non-covalently, can heal by only compression without heating (reproduced with permission from: Ref. [58], ©2018 AAAS)

While the addition of UPy hydrogen bonding has shown to be an effective way to achieve high stretchability, toughness and self-healing, the presence of a large number of hydrogen bonds often leads to crystallization or clustering that results in a brittle material [56, 57]. This has been addressed through the use of thiourea moieties as shown in Fig. 6 [58]. The key advantages of such thiourea moieties include the fact that they anomalously form a nonlinear zigzag thiourea hydrogen array, giving the strained trans/trans and adopting cis/trans conformations (Fig. 6I). On the other hand, at the latter stage, linear H-bonding urea units are formed by adopting only a trans/trans conformation (Fig. 6II), these two conformers (cis/trans and trans/trans) likely coexist in the polymer matrix of poly(ether-thioureas) with triethylene glycol (TUEG_3_). Furthermore, the tetraethylene glycol (TEG) spacers connecting thiourea arrays optimally modulate the activation energy (*E*_a_) of poly(ether-thioureas) with diethylene glycol (TUEG_2_), TUEG_3_, and poly(alkylene-thioureas) (TUC_8_) for the exchange of thiourea H-bonded pairs via slip motion. As a consequence, these polymeric materials, although highly crosslinked non-covalently, enables the fractured surfaces to be mechanically rejoined within reasonable time scales [58].

## Metal-containing supramolecular self-healing

Remarkable progress has been made in the field of metal-coordination bonds within metal-containing polymers over the previous two decades [43, 59]. These metal-containing polymers involve a combination of non-covalent binding between organic–inorganic networks that vary from strongly irreversible to highly dynamic [59, 60]. However, the latter has been attractive for self-repairing metal-containing polymers, offering reversibility similar to H-bonds [61]. The binding between metals and organic polymers can be adjusted by reversible bonds of low to high dissociation energies, which offer tunability and controllable self-repairing properties to the macromolecules by incorporating different metal ions and ligands substitutes [59]. Table 1 summarizes recent self-healing metal-containing supramolecular polymers, specifying their mechanical characteristics, healing motifs, and healing conditions.

**Fig. 7:**
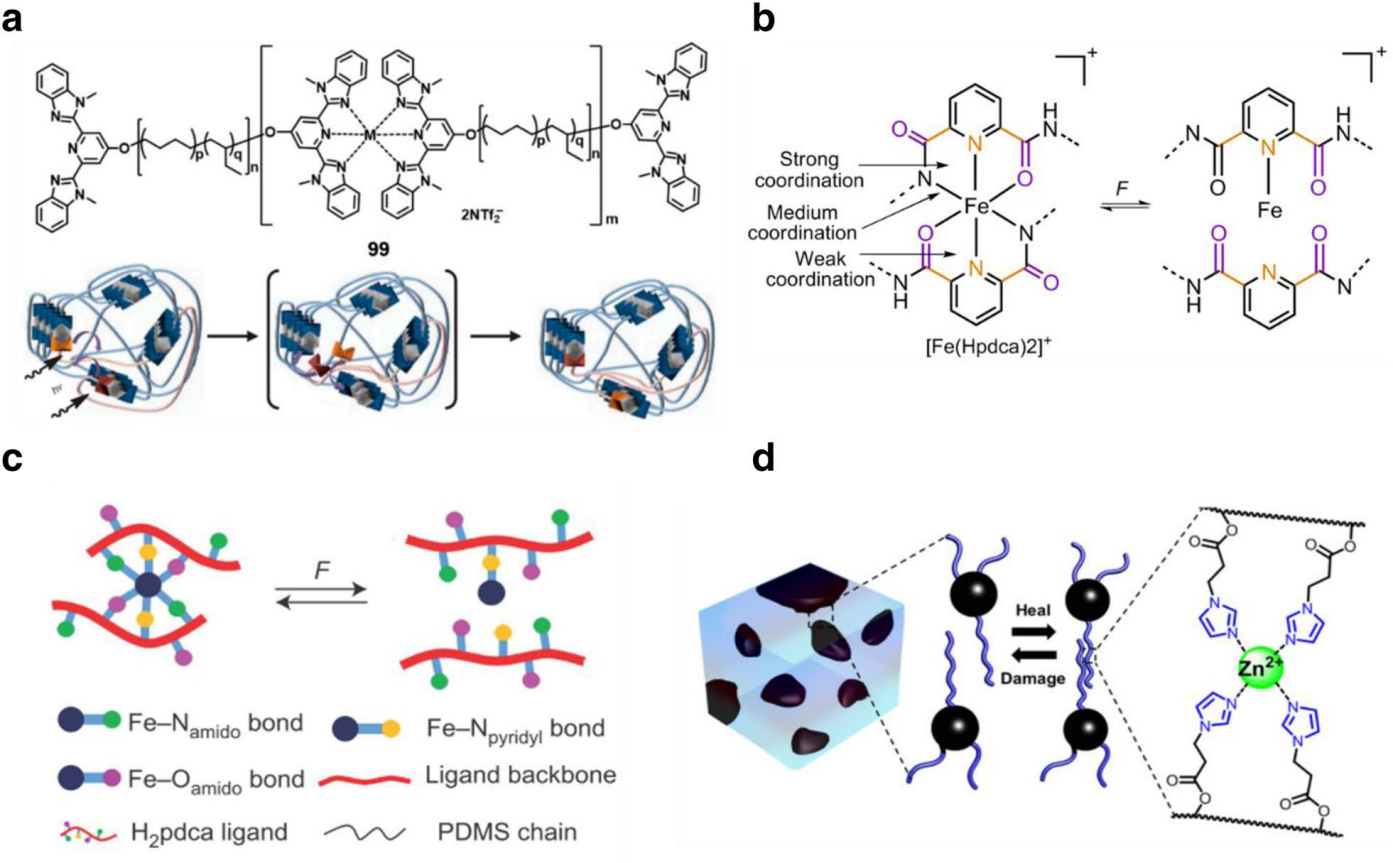
**a** Mechanism and synthesis of phase segregated healable metallosupramolecular polymers via assembly of 2,6-bis(1-methylbenzimidazolyl)pyridine (Mebip) ligands coordinated with Zn(NTf)_2_; **b** chemical structure and **c** schematic illustration of PDMS functionalized with 2,6-pyridinedicarboxamide ligands that coordinate to Fe(III) centers and the resulting Fe(III)–N_pyridyl_, Fe(III)–N_amido_ and Fe(III)–O_amido_ coordination bonds. **d** Chemical structure and microphase-separated soft\hard two-phase polymer system with metal–ligand interactions as the dynamic motif (figures reproduced with permission from: **a** Ref. [62], ©2011 NPG; **b** and **c** Ref. [28], ©2016 NPG; **d** Ref. [63], ©2014 ACS)

As shown by Burnworth et al. [62], self-healing can be achieved through a series of photoresponsive metallosupramolecular networks based on amorphous poly(ethylene-co-butylene) core with bifunctional 2,6-bis(19-methylbenzimidazolyl)pyridine (Mebip) ligand end-groups coordinated with metal ions Zn^2+^ or La^3+^ that are mended upon exposure to light (Fig. 7a). When expose to 320–390 nm UV light for 30 s, the damaged site of the metal–ligand complexes is electronically excited where the absorbed energy is transformed into heat. This makes depolymerization of the metal–ligand motifs and the decrease of the viscosity, thereby allowing healing of mechanical damages.

To achieve autonomous self-healing, Li et al. [28] designed an extremely stretchable and room-temperature self-repair polymer that utilizes metal coordination complexes with different bond forces that acted as crosslinks between the PDMS polymer chains (Fig. 7b). The coordination complexes consist of 2,6-pyridinedicarboxamide ligands that coordinate to Fe(II) centers resulting in three different interactions: a strong pyridyl–iron (Fe(III)–N_pyridyl_), and two weaker carboxamido-iron (Fe(III)–N_amido_, and Fe(III)–O_amido_) (Fig. 7b). Here, the inclusion of these weaker interactions allows energy dissipation upon stretching and self-healing when damaged, while the stronger interactions ensures that the metal ions are within the vicinity of the ligand for rapid self-healing to occur (Fig. 7b). Based on these modifications, the resultant films displayed outstanding self-healing abilities from room temperature to − 20 °C, where cut specimens could achieve a healing efficiency of up to 90% within 48 h at room temperature.

To complement autonomous self-healing with good mechanical strength, Mozhdehi et al. [63] strategically programmed a metal–ligand network within a multiphase polymer. In this work, a hard/soft two-phase brush copolymer system was designed whereby metal–ligand (zinc-imidazole) interactions were programmed within the soft phase for self-healing to be achieved. On the other hand, the hard phase comprised of glassy polystyrene with many imidazole-containing brushes (Fig. 7d) that allowed good mechanical properties to be obtained. In this system, dynamic and mechanical properties of the material can be conveniently adjusted through tuning a number of molecular parameters such as brush density, degree of polymerization, and ligand density. Furthermore, the benefits of such effective dynamic interactions within this hard/soft two-phase brush copolymer system could be observed through the perfect restoration in toughness and Young’s modulus as specimens were cut and healed under room temperature conditions for 3 h.

**Fig. 8:**
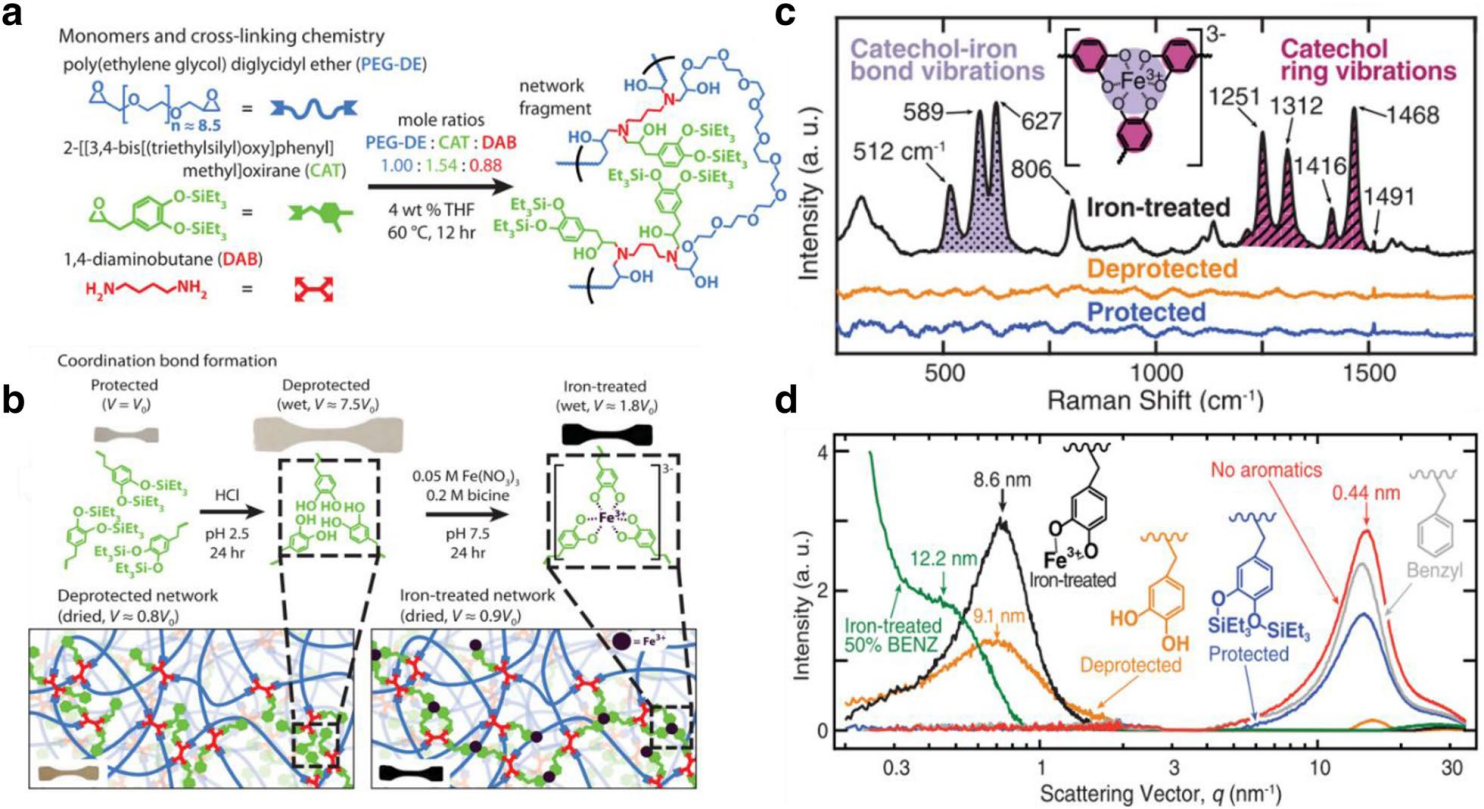
**a** Synthesis of catechol-containing networks and a triethylsilyl protected catechol group (CAT) cluster network-formation chemistry; **b** schematic representation of the silyl protective group’s cleavage and subsequent iron complex formation and a test specimen swelling during the process; **c** Raman spectra of protected (blue), deprotected (orange), and iron-treated (black) catechol-containing networks, which is confirm iron-catechol coordination; **d** SAXS and WAXS of the dry networks. SAXS shows broad peaks for the deprotected (orange) and iron-treated (black) networks. WAXS peaks result from interatom matrix scattering (reproduced with permission from: Ref. [64] ©2017, AAAS)

Another approach for toughening these self-healing elastomers is through the inclusion of iron–catecholate complexes [9]. This toughening effect has been demonstrated by Filippidi et al. [64] who fabricated an epoxy structure that behaved as a small series of bisepoxide [poly(ethylene glycol) diglycidyl ether (PEG-DE)], a monoepoxide leading a triethylsilyl preserved catechol group (CAT), and a tetrafunctional diamine cross-linker [1,4-diaminobutane (DAB)] (Fig. 8a, b). Owing to the incorporation of iron-catechol coordination bonds, significant improvements to the stiffness and toughness, including a 770-times improvement in elastic modulus, 92-times increase in toughness, 76-times development in yield stress, and a 58-times addition in tensile strength were being observed. This was further justified by wide-angle x-ray scattering (WAXS) and Raman spectroscopy (Fig. 8c, d), which identified the presence of these iron-catechol coordination bonds in the system.

Zhang et al. [65] introduced new dual-dynamic crosslinking substrate materials, which possess a combination of metal-coordination bonds (*β*-diketone–europium interaction) and hydrogen bonding, to enable multiphase separated network and inter-chain crosslinking. While sextuple Eu-curcumin coordinated bonds are typically very strong, they severely suppress molecular chain mobility that contributes to a moderate self-healing ability (90% for 48 h). Also, the influence of hydrogen bonds on the self-healing ability of materials is greatly limited because of the low hydrogen bonding sites (N–H and C=O).

One strategy to improve the mechanical strength of self-healing materials with high healing efficiencies is demonstrated by Wang et al. [66] who developed a robust metal–diamidepyridine (DAP) crosslinked elastomer from low-cost, commercially available compounds. Here, PU-DAP was synthesized by solvent-free polycondensation of poly(tetrahydrofuran), hexamethylene diisocyanate, and 2,6-diaminopyridine and the strength of coordination bonds is tuned through incorporating multiple metal-DAP motifs. When diamidepyridine polymers were crosslinked by ferric chloride (FeCl_3_·6H_2_O) (PU-DAP/Fe) or terbium trifluoromethanesulfonate (Tb(OTf)_3_) (PU-DAP/Tb), its high tensile strengths (up to 12.7 MPa) and strains (up to 1000%) could be completely restored after 24 h healing at 60 °C. In contrast, when the same polymer was crosslinked by zinc trifluoromethanesulfonate (Zn(OTf)_2_) (PU-DAP/Zn), self-healing was not observed due to the presence of the inert Zn-DAP bond, highlighting the effects of multiple metal-DAP motifs.

## Self-healing supramolecular polymer nanocomposites

Numerous studies have consistently demonstrated the significant impact of inorganic nanoparticles-based materials for light manipulation, microelectronics, energy harvesting and storage, and life sciences [67]. Through the co-assembly of inorganic nanoparticles with organic building blocks, polymer nanocomposites hold a variety of promising properties, such as hydrophilicity, hydrophobicity, and mechanical toughness [68]. However, despite the wide range benefits provided by these inorganic nanofillers, these polymer nanocomposites exhibit critical drawback. These nanofillers or nanoparticles introduced within the polymer may act as stress concentration points that cause polymer nanocomposites to be prone towards delamination fractures and micro-crack formation during transportation and application [69]. To address this limitation, studies on supramolecular self-healing of nanocomposite materials have been undertaken [70]. With the introduction of supramolecular self-healing to polymer nanocomposites, high-performance multifunctional polymers can be significantly enhanced.

To achieve the synergy between the supramolecular polymer matrix and its inorganic components is not a simple feat. Detrimental effects on the performance may arise from the interaction between the polymer matrix and the nanoparticles. Such effects include, (1) the formation of nanofiller networks that causes a gradual change of the polymer nanocomposites performance as it deviates from a standard Maxwell fluid to a viscoelastic solid, (2) the nanoparticle-polymer interaction that slows down the healing process or blocks it entirely by decreasing the density of reversible cross-links within the polymer matrixes; (3) the nanoparticle-polymer interaction that may soften and ultimately liquefy the composite in some particular cases, due to strong polymer–polymer crosslinks that are widely replaced by weak nanoparticle-polymer hydrogen bonds [67, 71].

The ability of supramolecular polymer nanocomposites to self-heal depends on the intrinsic ability of these heterogeneous materials to promote chain mobility and dynamic bond reordering [72]. Nanofillers such as nanotubes, nanofibers, hard nanoparticles within composite materials will not self-healing unless all components have naturally built-in segments of self-healing. Nevertheless, the interphase between the polymers and strengthening stiff inorganic particulate show the strength of C–C bond breaking and restoration, interfacial regions that may help the strengthening and toughening behavior of nanocomposites. The majority of nanorods or nanowires, nanoparticles, nanotubes, graphene and nanofibers, nanosheets can be surface treated to produce carboxylic acid [73], hydroxyl [74], UPy [75], thiol [76], amine [77], cinnamoyl [78], furfuryl [79], pyrenyl groups [80], anthracene [81] which can respond with their equivalents in the polymer matrix. Table 2 shows the recent self-healing methods based on supramolecular H-bonds and metal-coordination crosslinking: mechanical characteristics, self-healing conditions, and healing efficiencies.

**Table 2:** Summary of recent self-healing systems based on supramolecular hydrogen bonds and metal-coordination crosslinking: mechanical characteristics, self-healing-conditions and efficiencies

Material composition	*T*_g_ (°C)	Healing motif, condition, and efficiency	Remarks	Ref.
MG-SHPU	− 4.45	H-bonding, NIR radiation, and modulus of toughness, 40%	0.75 wt% MG (MG075) gave maximum healing efficiency up to 40%. Intermolecular diffusion of SHPU which was accelerated by thermal energy generated by NIR absorptions	[106]
RGO-HPUs	NA	H-bonding, MW energy and app. 100%	Composite is also incorporated with iron oxide/TiO_2_. This combined composites exhibited an excellent shape memory, self-healing and self-cleaning properties	[107]
		H-bonding, direct sunlight and NA		
G-PAM-PAA	NA	H-bonding, thermal healing and app. 100%	Above 10 wt% of graphene yields the self-healing behavior	[84]
GO-HBN	− 5 to 9	H-bonding, RT, ~ 60%	Protecting barrier for electronic wires and devices, sealing layer for gas systems	[102]
FG-TPU	~20–30	H-bonding, IR and MW radiation, and ~ 98%	FG-TPU composites exhibit improved mechanical properties and could be healed effectively and rapidly by IR, electricity, and electromagnetic wave	[84]
µNi-M-NH_2_	− 20 to 10	H-bonding, RT, 15 min, ~ 90%,	Addition of nanostructured µNi particles with nano-structured surfaces significantly enhances the mechanical properties	[108]
BNNSs-supramolecules	− 7 to 43	H-bonding, ~ 100%, 85 °C, 30 min	Polymer nanocomposite with 8 vol% BNNSs exhibits two orders of magnitude improvement in electrical resistivity over that of the pristine polymer network	[109]
UPy-K-UPy/CNC-UPy	− 50	H-bonding, UV-light, 20–80 s, ~ 100%	H-bonded UPy supramolecular polymer and CNCs decorated with the same supramolecular motif show an attractive combination of high stiffness, high strength, and rapid and efficient optical healing	[75]
Si-GO-HPU	− 50 to − 51	H-bonding, UV-MW, 4–6 min ~ 100%	The surfaces of the HPU/Si-GO nanocomposites also displayed inherent hydrophobicity without any additional surface modification	[110]
CNT-PU	NA	Coordination bond, NIR-light, 90 °C, 1 h, ~ 93%	Zn^2+^ coordinated metallo-supramolecular CNT-PU nanocomposite that showed a strong, tough, and elastic mechanical properties and was able to self-heal multiple times	[89]
V_2_O_5_-PDMS-g-PUR	− 12.5 to − 53.1	H-bonding, 50 °C, 120 min, 85.4%	V_2_O_5_ nanofibers enhances the mechanical properties and healing efficiency of the PDMS-g-PUR through a reversible hydrogen bonding mechanism	[111]
GO-PDMAA	NA	H-bonding, NIR radiation, 3 min, 96%	Self-healable GO-clay-PDMAA hybrid hydrogels with high extensibility and mechanical strength contributed by both hectorite clay and GO as cross-linking agents	[112]
GO-PAA	NA	H-bonding, NIR radiation, 30 °C, 24 h, ~ 100%	The strong interactions between the PAA chains and the GO sheets are essential to the mechanical strengths of the healed gels	[113]
GO-PAACA		Coordination bond and H-bonding, Ph7, 10 min, ~ 100%	The polar groups of the PAACA side chains and oxygen-containing groups of GO nanosheets via coordination interactions	[114]
HPU-SRGO	NA	H-bonding, 50-60 s in MW, 1–3 min in sunlight, ~ 96.8%	Shape memory of HPU and energy absorbing capability of SRGO-assisted melting, diffusion, and rearrangement of the soft segment of HPU to crack place	[115]

SHPU-MG: modified graphene (MG)/self-healable polyurethane (SHPU) nanocomposites; NIR radiation: near infrared (NIR) light absorption; RGO-HPUs: reduced graphene oxide (TiO_2_/RGO)/hyperbranched polyurethane (HPU)-TiO_2_ nanocomposite; MW energy: microwave power direct sunlight; G-PAM-PAA: graphene modified with poly(acrylamide-co-acrylic acid); GO-HBN: graphene oxide (GO)-amine terminated oligomer (HBN); FG-TPU: few-layer graphene (FG)-thermoplastic polyurethane (TPU); IR-MW: IR radiation electrical simulation and microwave radiations; µNi-M-NH_2_: nickel-randomly branched oligomer (M-NH_2_); surface functionalized boron nitride nanosheets (BNNSs); hyperbranched polyurethane (HPU)-aminopropyltriethoxysilane-modified graphene oxide sheets (Si-GO) V nanocomposites; CNT-PU: terpyridine ligand-terminated CNT/PU prepolymers; self-healing polydimethylsiloxane-graft-polyurethane (PDMS-g-PUR)/vanadium pentoxide (V_2_O_5_) nanofiber supramolecular polymer composites; GO-PDMAA: graphene oxide (GO)-hectorite clay-poly(*N*,*N*-dimethylacrylamide) (PDMAA); GO-PAACA: graphene oxide (GO)/poly(acryloyl-6-aminocaproic acid) (PAACA); HPU/SRGO: hyperbranched polyurethane (HPU)-sulfur nanoparticles decorated reduced graphene oxide (SRGO) nanocomposites

### H-bonded self-healing supramolecular polymer nanocomposites

**Fig. 9:**
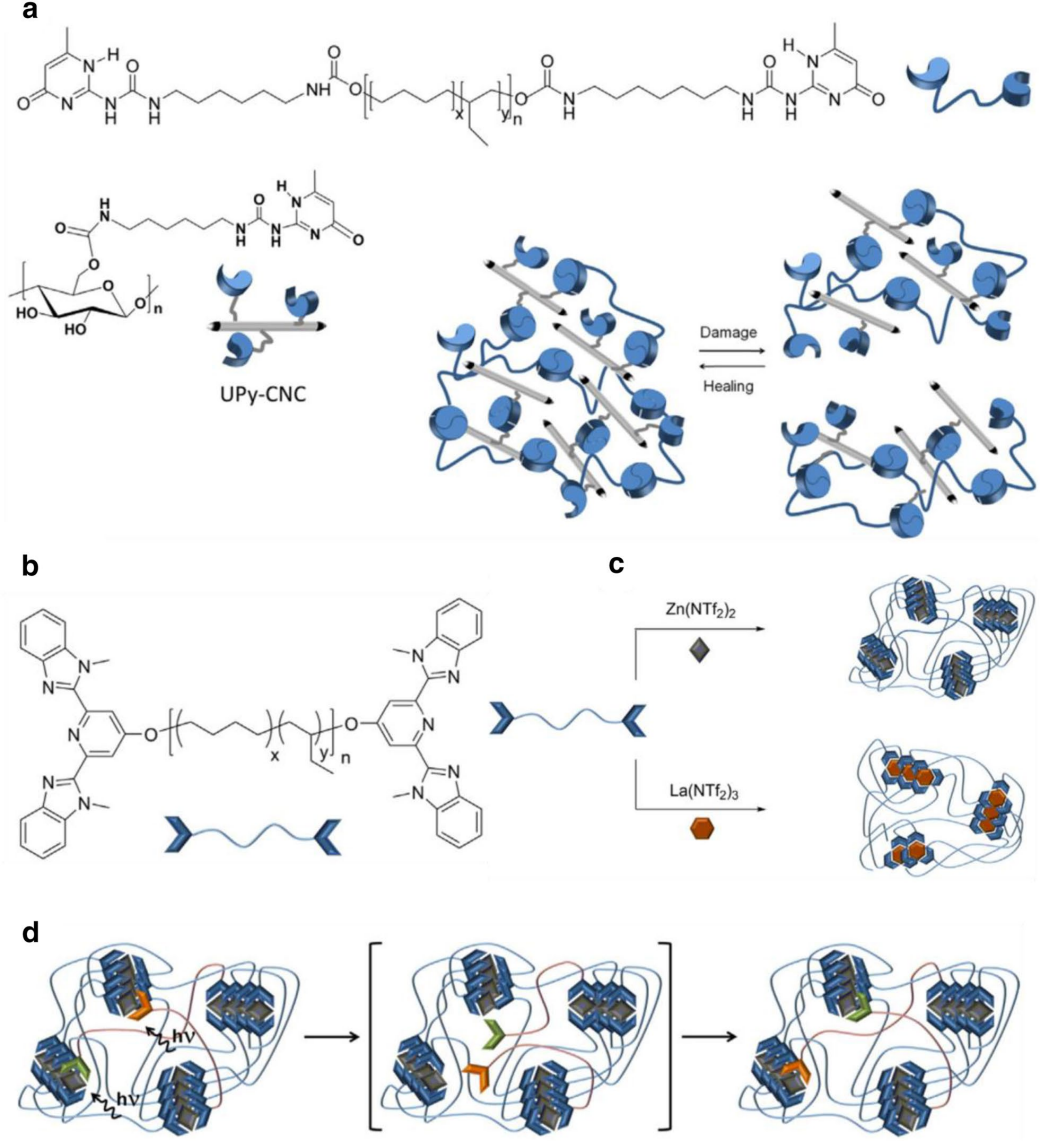
**a** Chemical structures and schematic illustration of CNC functionalized self-healing of UPy nanocomposites based on telechelic PEB building blocks. **b**, **c** Chemical structure of the metallopolymers via assembly of Mebip end-capped telechelic poly(ethylene-co-butylene) with Zn(NTf_2_)_2_ or Eu(NTf_2_)_3_. **d** Schematic representation of the UV-light triggered metallosupramolecular nanocomposites (figures reproduced with permission from: **a** Ref. [75], ©2013 ACS; **b**–**d** Ref. [92], ©2011 NPG; **b**–**d** Ref. [93], ©2013 ACS)

As shown in Fig. 9, the light-healable nanocomposites composed of a telechelic PEB that was functionalized with hydrogen-bonded UPy and reinforcing cellulose nanocrystals (CNC) fillers. These fillers carried numerous OH groups that interact with UPy-NCO to make tough UPy–UPy H-bonding interplays with the same supramolecular motif [68]. Films with deliberately introduced defects were healed in a matter of 80 s under UV absorption at 320–390 nm of wavelength with 250–350 mW/cm^2^ of intensity, at a content of 20 (wt%) of CNC-UPy. The bio-derived supramolecular nanocomposites with the special CNC-matrix and CNC–CNC interplays could increase the tensile storage modulus improved of 10 MPa for the pure polymer to 247 MPa for the composites containing 20 (wt%) CNC. Hence, this light-healable nanocomposite exhibited an attractive order of mechanical properties, homogeneity and efficient healing behavior. Inspired by this concept, noble metal nanorods that possess unusual optical properties were explored as well. These superior metal nanorods may possibly be functionalized with photo-responsive working assemblies (anthracene, cinnamoyl, and UPy) to fabricate composites with optoelectronic properties and remotely controlled healing performance [82, 83].

**Fig. 10:**
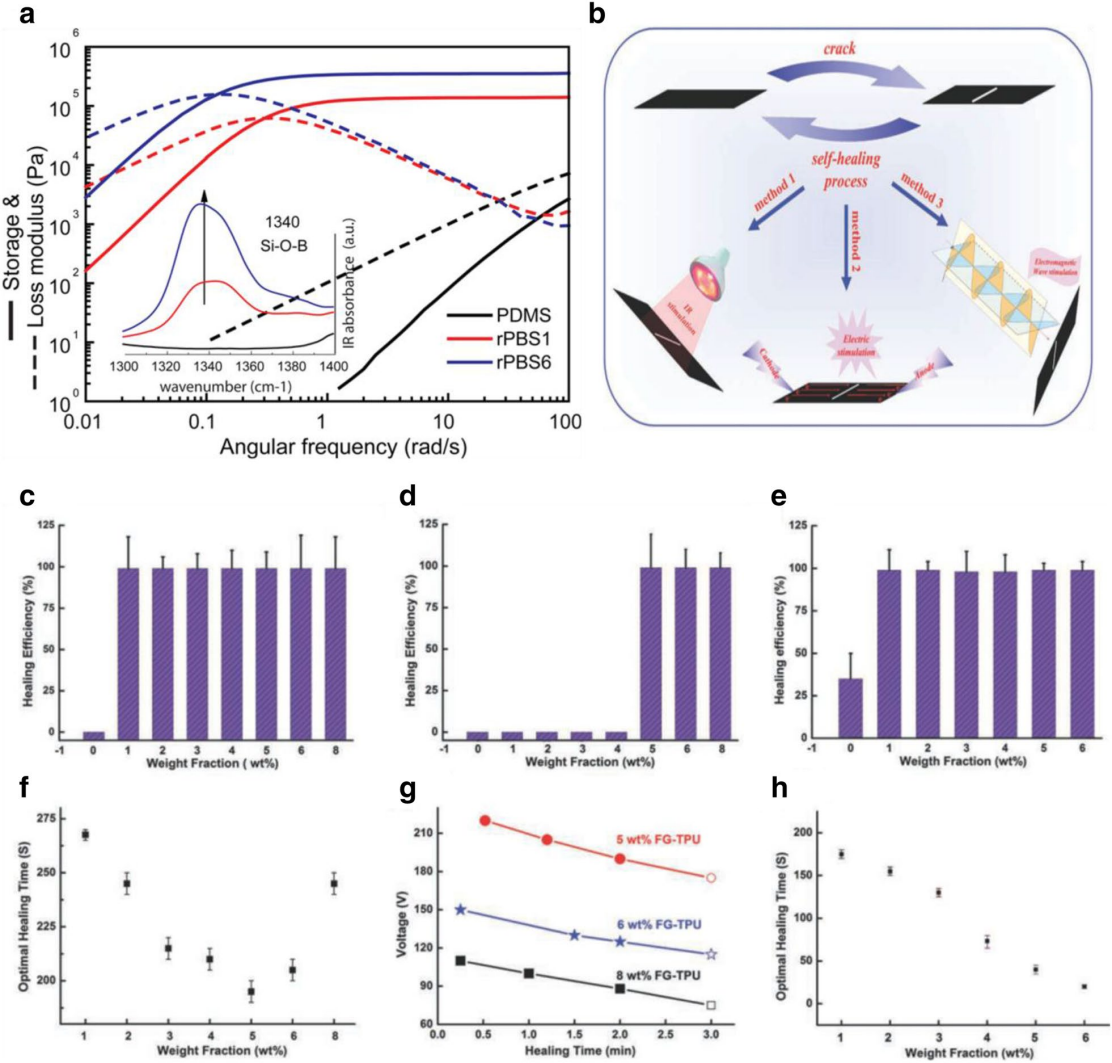
**a** The dynamic storage (G′) and loss (G″) moduli of PBSs of different reaction time, measured at 20 °C; **c** the IR light healing efficiencies; **b** the healing performances of FG-TPU composites were healed by IR light, electricity, and electromagnetic wave; **d** the electrical healing efficiencies; **e** the electromagnetic wave healing efficiencies; **f** the optimal healing time; **g** the relationship between the applied voltage and the healing time; **h** the optimal healing time of the FG-TPU samples (figures reproduced with permission from: **a** Ref. [85], ©2014 ACS; **b**–**h** Ref. [84], ©2013 WILEY)

Huang et al. [84] fabricated a new self-repair few-layer graphene (FG)-thermoplastic polyurethane (TPU) FG-TPU nanocomposite. These FG-TPU nanocomposite exhibits enhanced mechanical properties and could be repaired efficiently and repeatedly without degradation to its healing efficiencies. This FG-TPU self-healing nanocomposite can be healed via various processes including electricity, electromagnetic wave and infrared light. As shown in Fig. 10b, a healing efficiency of above 98% was achieved in all three cases. This exceptional self-healing ability of the nanocomposite can be associated with the energy-absorbing capacity and the large thermal conductivity of graphene. As such, the energy from all three origins could be effectively transferred into the TPU matrix, where the energy enabled TPU chains at the interface to spread and re-entangle to retain its properties. From Fig. 10c–h, the effects of graphene on the healing efficiency of FG-TPU composites can be clearly demonstrated through the various healing efficiency achieved at different loadings amount of graphene.

Through melting a compound of PDMS and boric acid (BA) up to 200 °C, Besseling et al. [85] synthesized polyborosiloxanes (PBSs), a supramolecular elastomer with hydrogen bonding between end groups. This modification of PDMS to PBSs was also verified from the addition of the molecular weight distribution, and the contemporary production of –Si–O −(BO)_m_(OH)_n_ moieties. Also, the noticeable increment of the mechanical moduli upon adjustment of PDMS by BA was attributed to H-bonds within BA based groups of the PBS. As these H-bonds are reversible and are easily renewed upon fracture, PBS displayed intrinsic self-healing capacities. Thus, both little cracks and obvious failures of PBSs could be healed without external stimuli. These supramolecular elastomers have been used for several purposes, such as silly putty, bouncing putty [86], dilatant compounds [87], etc. Figure 10a, the viscoelastic response of the supramolecular elastomers showed a dramatic improvement with an increase in the dynamic moduli (G′) at − 100 °C. This shows that the *T*_g_ of PBSs was significantly enhanced as opposed to virgin PDMS, shifting from − 120 to − 150 °C.

### Coordination bonded self-healing supramolecular polymer nanocomposites

As an alternative to H-bonds based nanocomposites, self-healable nanocomposites based on CNCs-reinforced metallosupramolecular polymer (MSP) were recently reported (Fig. 9) [62]. The materials were synthesized by the association of Zn(NTf_2_)_2_ and telechelic PEB that was end-functionalized with 2,6-bis(1′-methylbenzimidazolyl)pyridine (MeBIP) ligands. This surface unmodified CNCs can only be well dispersed in protic polar solvents such as water and DMSO [88], which do not permit dissolution of the metallosupramolecular polymer. Upon UV light irradiation, the metal complex generates heat, causing the temporary dissociation of metal–ligand motifs. As a result, the material begins to liquefy, allowing it to readily fill small defects. When the radiation is turned off, the metallopolymer reassembles the restoration of its original properties. The initiation of CNCs inside the metallosupramolecular leads to a notable improvement in the stiffness and strength, from 52 and 1.7 MPa for the neat MSP to 135–5.6 MPa upon incorporation of 10 wt% CNCs. It was anticipated that the surface hydroxyl groups of the CNCs were bound to the Zn^2+^ ions, thereby connecting the reinforcing filler with the MSP matrix. The damaged specimens of these metallosupramolecular nanocomposites were demonstrated to have efficiently restored next 30 s of exposure to UV-light.

Zheng et al. [89] developed a novel terpyridine ligand-terminated CNT/PU prepolymer that was dynamically crosslinked with metal ion Zn^2+^ to obtain a multi-stimuli responsive self-healing metallosupramolecular nanocomposite. From this modification, the resulting metallosupramolecular CNT/PU nanocomposite displayed outstanding mechanical properties where the tensile strength, strain-at-break, and toughness of the metallosupramolecular polymer nanocomposite films increased from 14.2 MPa, 620%, and 51.4 MPa to 22.8 MPa, 1076%, and 141.2 MPa, respectively. In addition, multiple stimuli self-healing was obtained via remotely controlled near infrared (NIR) light, low temperatures or solvents with excellent healing efficiencies and short healing times. Therefore, by combining self-healing capabilities with superior mechanical properties, metallosupramolecular nanocomposite offers many promising applications including soft electronics, nano/microelectronic, sporting equipments, and structural components.

## Conclusions and future perspectives

In a nutshell, we have highlighted the burgeoning trend of utilizing supramolecular association and re-association interactions for self-healing polymeric systems. From the examples discussed in this review, self-healing supramolecular polymers based on either H-bonding or coordinative bonds, have been demonstrated to be highly advantageous and versatile for next-generation self-healing materials capable of undergoing multiple healing cycles. The incorporation of supramolecular non-covalent crosslinks such as hydrogen bonding is the key component over the formation of associative groups and a wide range of responsive materials and dynamics. However, there remains to be a balance and trade-off between the network structures and bond dynamics properties. While a large number of self-healing concepts and approaches were introduced to describe the applications of these materials, there is still a need for a deeper understanding of the principles of self-healing and its basic mechanisms to address the many considerations in real-world applications.

Adopting supramolecular polymers for self-healing applications remain highly challenging due to a multitude of factors. This includes the formation of crystallization aggregation, interfacial effects, clustering, and phase segregation. Nonetheless, it should be noted that the preparation of self-healing materials and composites for specific purposes are still in its nascent stage. Most of the reported self-healable materials require activation under different conditions (light, temperature, vacuum, humidity, pressure, etc.). In addition, the well-known issue of hard supramolecular materials that arises from the incompatibility of dynamic supramolecular healing with low chain mobility to achieve high deformability remains to be a challenging task. Attaining effective healing over a short time span remains a critical need in the field of self-healing polymers.

Several exciting breakthroughs have been made under the endeavors of intensive research. Major innovative approaches for new self-healing supramolecular soft materials are anticipated in the near future. Undoubtedly, the application of self-healing supramolecular soft materials is the impetus for the advance of this field. At this juncture, supramolecular polymers based on multiple hydrogen bonds and metal-coordination bond have had a strong impact on materials science. Developing self-healing and smart responsive materials remains to be a promising domain to extend and inject new vigor into this emerging research field of supramolecular soft materials.

## Data Availability

Not applicable.
